# Determinants of financial inclusion in households in Peru

**DOI:** 10.3389/fsoc.2024.1196651

**Published:** 2024-02-16

**Authors:** Julio Cesar Quispe Mamani, Santotomas Licimaco Aguilar Pinto, Dominga Asunción Calcina Álvarez, Marleny Quispe Layme, Guino Percy Gutierrez Toledo, Gina Tamara Condori Condori, Luis Vargas Espinoza, Wilian Quispe Layme, Hugo Rubén Marca Maquera, Charles Arturo Rosado Chávez

**Affiliations:** ^1^Faculty of Economic Engineering, National University of the Altiplano, Puno, Peru; ^2^Faculty of Administrative Sciences, Andean University Nestor Caceres Velasquez, Juliaca, Peru; ^3^Academic Department of Education and Humanities, Faculty of Education, Amazon National University of Madre of Dios, Puerto Maldonado, Peru; ^4^Education Faculty, Amazon National University of Madre of Dios, Puerto Maldonado, Peru; ^5^Faculty of Accounting and Administrative Sciences, National University of the Altiplano, Puno, Peru; ^6^Faculty of Business Sciences, Peruvian Union University, Juliaca, Peru; ^7^Faculty of Administrative Economic Sciences, Catholic University of Santa Maria, Arequipa, Peru; ^8^Ecotourism Faculty, Amazon National University of Madre of Dios, Puerto Maldonado, Peru; ^9^Professional School of Public Management and Social Development, National University of Moquegua, Moquegua, Peru

**Keywords:** inclusion, economic income, education, financial system, household

## Abstract

The issue of financial inclusion considers access to and use of quality financial services by household members and different types of companies around the world, allowing us to reach the opportunities that the globalized world offers us. The objective of this research was to identify the socioeconomic factors that determined the inclusion of households in the financial system in Peru in the period of 2021. A quantitative approach was considered, which was non-experimental with a descriptive and correlational design and in which 81,441 pieces of data were obtained from the National Household Survey (ENAHO) of the National Institute of Statistics and Informatics, applying a logit binomial regression. It was determined that 47.02% of households were included in the financial system; 61.93% of those surveyed had their residence in the urban area; on average, respondents had incomplete secondary education; the age of the respondents on average was from 25 to 44 years; the average economic income of the household was less than $251 per month; 72.18% were represented by men as heads of the household and the rest by women; most of the respondents had a cohabiting marital status; the social conditions showed that 23.82% were in the group of being poor; and the majority of households did not have a property title. The determinants of financial inclusion in Peruvian households for 2021 were the area of residence, educational level, age of the respondent, economic income, gender of the respondent, marital status, social status, and property title.

## 1 Introduction

At the global level, financial inclusion is a fundamental factor for the functioning of the world economy, and it is also very important, given that a country with high financial inclusion will be able to improve the economic and social growth of its most vulnerable citizens who have lower incomes, thus improving their quality of life and boosting economic activity (Galor and Zeira, 1993; Robert et al., 2013; Datta and Singh, 2019; Suresh et al., 2022). However, one of the main aspects of the current world economic panorama is the growing importance of financial markets at the international level, in which the majority of residents of different countries trade assets, shares, bonds, and financial instruments, as well as bank deposits denominated in different currencies (Stiglitz, 2009; Arun and Kamath, 2015; Huang et al., 2022; Zeraibi et al., 2023).

The aforementioned point is corroborated by Cermeño and Roa (2013), who established that most economies showed an average of 19% of people having a current credit, with a sustained growth in the number of points to carry out transactions, especially of automated teller machines (ATMs). However, there is a significant delay in the levels of access with respect to that of the most developed economies since only 65% were received in the formal financial sector.

Complementarily, there is some important evidence on financial inclusion that has showed important results; for example, Tuesta and Sorensen (2015), who established that the three dimensions that determine financial inclusion are access, since 50% of bank branches are in the capital; use, with was 47.8% receiving work or sale payments via bank branches; and barriers, in the case of Argentina. The authors Suárez and Pacheco (2017) developed the regulatory index to improve financial inclusion and compare with the Latin American countries Argentina, Brazil, Chile, Colombia, Mexico, Paraguay, Peru, and Uruguay, thus finding important results for Peru, since it was in the first position when it came to promoting credit with an index of 1.9. Finally, there was Argentina with 1.1, Brazil with 1.4, Chile with 1.25, Colombia with 1.75, Mexico with 1.35, Paraguay with 1.40, and lastly, Uruguay with 1.50.

In addition, other investigations have been conducted, such as that of Anaya-Narváez and Romero-Álvarez (2018), who demonstrated that there is an inverse relationship between monetary poverty and financial inclusion. The variable that most influences a household to have a greater probability of accessing the financial system is the educational level of the head of the household; in addition, if the household receives direct state aid and if the head of the household is a woman, the chances of financial inclusion are reduced. According to Cardona-Ruiz et al. (2018), being a woman in Colombia has a negative impact on financial inclusion. Taking into account the estimates made, it is seen that being a woman reduces the probability of an individual having an account in the financial system by 9.5 percentage points; the person having a debit card by 11.7 percentage points; a person having a debit card in her name by 10.7 percentage points; and the probability that the person uses a credit card by 3.9 percentage points (Imai et al., 2010; Arun and Kamath, 2015; Orazi et al., 2021).

Complementing the aforementioned, at the Latin American level, considering Mexico and other countries as a reference, it was determined that the level of financial inclusion reaches 38.3 percentage points for the evaluated country, which is a very low result compared to that of other continents. It is also displayed that women (35.9%), people over 60 years of age (29.6%), people from rural areas (31.7%), people with lower socioeconomic levels (31.4%), and people with lower educational levels (people who have a primary level have 35.9%) are the most financially excluded groups. Likewise, in Mexico, older adults have high financial knowledge (0.7749). In addition, it is confirmed that there is a significant relationship (*p* < 0.01) (Rodríguez-Raga and Rodríguez, 2016; Ferraz and Ramos, 2018; Grupo Crédito, 2022).

At the Peruvian level, in 2019, 44.9% of people in the Economically Active Population (EAP) and in an employed situation (people who work in the formal and informal sector) had access to a payment card or account, which shows that less than half of the employed were included in the financial system. In this regard, it is observed that inclusion is greater in the group of people aged between 25 and 40 years old (50.5%), people who have a university education (79%), and people who live in urban areas (49.2%). Likewise, it is observed that the lowest levels of financial inclusion occur in the group of people aged 56 and over (38%), people with primary education (24%), and people who live in rural areas (26%). According to INEI (2020), the number of people who have an account in the financial system increased from 43.4% of the total population in the last quarter of 2019 to 49% in the last quarter of 2020.

That is why Lahura and Alonso (2016), as of December 2014, found that 19% of all debtors had at least one loan in some entity of the financial system, which indicates that there would be a significant margin for expanding the tax base. It was also found that the rural banks Edpymes and Financial are the entities with the highest rates of informality. In addition, the loans obtained by the informal banks were granted mainly by financial institutions, municipal savings banks, and banks, while the most important amounts correspond to loans for micro and small businesses (Mypes), consumer loans, and credit cards. Moreover, the tax evasion of informal clients of the financial system would have been approximately 0.7% of the GDP in the year 2014, for which both quantitative and qualitative methods were used, and this is specifically based on the four most representative banks of the financial system, with variables such as corporate reputation (through the Merco index value) and financial solvency taken from the companies’ reports (2011–2014). Managing to demonstrate that the reputation of each mentioned bank is very important because it has a positive impact on the financial results of banks in the local financial system, and this reputation would also have an effect on economic solvency.

One of the results that is closely related to financial inclusion in Peru is the one developed by Cámara et al. (2013), who estimated a series of probit-type models that allowed them to analyze the correlations between financial inclusion and some explanatory variables. The variables taken into account were possession and use of formal financial products, geographical area, being a woman, marital status, literacy, annual spending, income, age, educational level, savings level, household indebtedness level, level of annual spending on mobile phones, and the number of population centers. They used information from the National Household Survey (ENAHO) of the year 2011, and it was concluded that those groups were recognized as more vulnerable; that is, women, inhabitants of rural areas, and young people are the ones who have the greatest difficulties in entering the formal financial system.

In this sense, since there is asymmetry on the part of the people who influence their financial inclusion, it is important to answer the following question: What were the socioeconomic factors that determined that families in the Puno region were included in the financial system in the year 2021?. The objective of the research was to identify the socioeconomic factors that determined the inclusion of households in the financial system in Peru in the period of 2021.

## 2 Literature review

### 2.1 Finance

Finance is the set of economic activities that are related to money, whether in business, banking, or the stock market, taking place in a group of markets or financial institutions of national or international scope (Padilla and Manuel, 2014).

### 2.2 Finance system

The financial system is “A set of institutions, instruments and markets through which savings are channeled toward investment (Joaquín and González, 2008)”.

### 2.3 Financial inclusion

Financial inclusion is the result of the interaction of factors that affect the demand for financial services by households and firms and their supply by financial institutions (Roa and Carvallo, 2018).

Financial inclusion in recent decades has gained significant relevance in the world, given that every day it is evidenced as an important tool that helps families, households, and society as a whole to boost economic prospects; therefore, this has positive consequences on the economic growth of a country, since it not only contributes to improving economic conditions but also the quality of life of its population (Jappelli and Pagano, 1994; Hassan et al., 2011; Li et al., 2013; Bruhn and Love, 2014; Bohl et al., 2015; Zeraibi et al., 2023). In addition, the use of financial services and products in these times has become very important for the private and public sector, since it makes it possible to dynamize the economy as a whole, generating greater opportunities among the individuals who participate, improving the optimal achievement of the objectives that people, companies, and organizations, among others, may have set. The products offered by financial services groups include savings loans, and insurance, among others (Loayza and Ranciere, 2006; Beck et al., 2007; Jeanneney and Kpodar, 2011; Dupas and Robinson, 2013; Robert et al., 2013; Rodríguez-Raga and Riaño-Rodríguez, 2016; Ferraz and Ramos, 2018; Kabakova and Plaksenkov, 2018; Orazi et al., 2019).

#### 2.3.1 Asymmetric information

Asymmetric information occurs when one participant in an economic transaction has more information relevant to said transaction than the other participant. This is why asymmetric information presents three problems (Wong et al., 2012):

- *The problem of moral hazard*

Moral hazard refers to situations in which one side of the market cannot observe what the other side is doing. For this reason, it is sometimes called the hidden action problem (Varian, 2015).

- *The problem of adverse selection*

Adverse selection is the type of market failure that will occur when products of different quality are offered at a single price thanks to a lack of information; that is why a greater number of low-quality products are sold, and on the other hand, too little quantity of good-quality products are sold (Varian, 2015).

- *The problem of herd behavior*

Herd behavior refers to the fact that a certain group of people imitate a crowd during a certain period, often not considering individual information that suggests following another path (Banerjee, 1992).

#### 2.3.2 Asymmetric information theories

##### 2.3.2.1 Credit rationing theory

This theory detects market failures caused by moral hazard and adverse selection as the root of credit rationing when there is asymmetric information. This lack of information leads to credit rationing, when the interest rate or size of the loan chosen alters the behavior of the borrower (moral risk), or the risks that occur when matching applicants (good and bad) to the credit (adverse selection). There is also the class of customer models with affinity, with the assumption that customers with the longest time have priority access to some credit; however, these models may also need asymmetric information to generate the distribution of credit (Sánchez-Daza, 2001).

##### 2.3.2.2 Portfolio theory

This theory, which began with Harry Markowitz in 1952, is based on plurality, which is the main concept of creating optimal portfolios, which are combinations of assets with the best risk–return relationships. This risk is evaluated by estimating the variance of the expected returns linked to the assets that are adjusted to it. On the other hand, diversification when investing in more than one asset aims to reduce the level of risk that is linked to different factors of the company, which, unlike investing in a single asset, would be less exposed. However, in any case, the risk would never be reduced to zero, since there are external factors that prevent it, so this exposure to risk makes it not diversifiable. For this, it is advisable to have a portfolio of prudent and easy-to-manage assets, and the correct number is one that, by adding an additional asset, means the risk reduction is not significant (Bejarano et al., 2013).

#### 2.3.3 Information asymmetry in inclusive financial markets

Addressing the issue of the presence of information asymmetries is broad. Therefore, Sánchez-Daza (2001) highlights the difference that must be made between uncertainty and asymmetric information, where in the first case it is oriented toward the existence of incomplete information and in the second case to highlight the non-availability of information in the market. In the area of microfinance, the existence of these two aspects is common. Since there are moral risks and adverse selection to which they can induce us, it is because of them that the operation of microfinance cannot achieve efficiency in the Pareto sense, which is also caused by the existence of externalities in transaction costs.

In addition, there is an imperfect financial market, which seeks to be inclusive but has as its breaking point a traditional financial system and, in some cases, high rates of informality. That is why asymmetry cannot be seen, much less quantified, while it occurs, but they it be perceived after it produces the effects of risk and uncertainty for both financial companies and users (Sánchez-Daza, 2001).

That is why, in finance, the existence of information asymmetry definitively alters the assumption of efficiency, the existence of risk neutrality, and the existence of optimizing behavior of agents whenever financial entities receive incomplete information on the solvency and credit quality of the user, which affects the credit evaluation process. That is why financial companies are suggested to approach it from a comprehensive perspective, guaranteeing the implementation of effective financial education, monitoring, and sustainability (Bejarano et al., 2013).

## 3 Materials and methods

### 3.1 Approach, type, and design of the investigation

The present investigation undertook a quantitative approach, of a non-experimental type, with a descriptive and correlational design because, in this situation, the variables were analyzed from their natural state, since there was no manipulation to be able to visualize, contrast, and verify their behavior. Likewise, the logit-type regression model was used, applying cross-sectional data from the year 2020 with data from the National Household Survey of the National Institute of Statistics and Informatics (INEI) (Waldo, 2014; Carlos and Sampieri, 2017).

### 3.2 Techniques and instruments for collecting information

A documentary review was carried out, which allowed for a review and compilation of information extracted from articles and books, referring to the research topic, with the purpose of selecting information to compare with the results of the work.

The data used were from a secondary source, namely, the database of the National Household Survey (ENAHO) of the National Institute of Statistics and Informatics (INEI). The processing was performed using the STATA 16.0 statistical program, which allowed us to perform a descriptive analysis and then perform the Logit model regression and the regressor selection tests necessary to validate our model.

### 3.3 Data

The population for this research was the number of people considered in the ENAHO database, who were registered by the INEI for the year 2021, amounting to approximately 1,237,997 people.

In addition, according to the INEI, the sample considered for the ENAHO survey for 2021 was of the probabilistic, area, stratified, multistage, and independent type in each study region at the level of Peru, where a confidence level of the sample results of 95% was considered. Moreover, to establish the determinants of financial inclusion of households in Peru, household members aged 14 years and above were considered, with defined socioeconomic characteristics and a maximum level of education achieved among those who have conditions of access to the financial system. The total sample for the study group was thus 81,441 observations.

### 3.4 Variables

The study variables for this research were obtained from the ENAHO-2021 database, which were financial inclusion, area of residence, level of education, age, family income, gender, marital status, social status, and property title (Table 1).

**Table 1:** Characteristics of the variables.

Variables	Indicator	Category	Data type	Measurement scale	Fountain
Dependent					
Financial Inclusion	Has access to the financial system	1: Yes 0: No	Qualitative	Nominal	INEI
Independent					
Area of residence	Household location	1: Urban 0: Rural	Qualitative	Nominal	INEI
Educational level	Level of education	1: No level 2: Initial 3: Incomplete primary 4: Complete Primary 5: Incomplete Secondary 6: Secondary complete 7: Incomplete non-university superior 8: Full non-university high school 9: Incomplete university high school 10: Full university high school 11: University postgraduate	Qualitative	Ordinal	INEI
Age	Life years	1: 18 to 24 years 2: 25 to 44 years 3: 45 to 64 years 4: 65 years and older	Quantitative	Discreet	INEI
Income level	Amount of monthly income received	1: Less than $251 2: From $251 to $502 3: From $503 to $753 4: From $754 to $1,005 5: S/1006 to $1,256 6: Above $1256.00	Quantitative	Continuous	INEI
Marital status	Marital status	1: Married 0: Single	Qualitative	Ordinal	INEI
Gender	Gender	1: Man 0: Women	Qualitative	Nominal	INEI
Social status	Level of social status	1: Poor 0: Not poor	Qualitative	Ordinal	INEI
Title deed	The household has title deed	1: Yes 0: No	Qualitative	Nominal	INEI

### 3.5 Approach to the econometric model

For the present investigation, the logit-type model was used, which is represented as follows:


ProbY=1=e^X^′β/1+e^X^′β

$$\begin{array}{l} \mathrm{Prob (Fin}\mathrm{ancia}\mathrm{l inclusion)}=1\\ \begin{array}{l} =e^{\Lambda }(\beta \_0+\beta \_1\mathrm{Area}\;\mathrm{of}\;res\mathrm{idence}+\beta_{\text{\_}}2\;\mathrm{Level of education}\;+\beta \_3\;{\mathrm{Respondent}}^{\Lambda }\mathrm{'s age}\\ +\beta \_4\;\mathrm{Family income}+\beta \_5\;{\mathrm{Respondent}}^{\Lambda }\mathrm{' s gender}\\ +\beta \_6\;\mathrm{Marital status}+\beta \_7\;\mathrm{Social status}+\beta \_8\;\mathrm{Title deed})\\ /(1+e^{\Lambda }(\beta \_0\;+\beta \_1\;\mathrm{Area of residence}+\beta \_2\;\mathrm{Level of education}\\ +\beta \_3\;{\mathrm{Respondent}}^{\Lambda }\mathrm{' s age}+\beta \_4\;\mathrm{Family income}+\beta \_5\;{\mathrm{Respondent}}^{\Lambda }\mathrm{' s gender}\\ +\beta \_6\;\mathrm{Marital status}\;+\beta \_7\;\mathrm{Social status}+\beta \_8\;\mathrm{Title deed)}) \end{array} \end{array}$$

## 4 Results

To determine whether there was financial inclusion, it was arranged to ask the respondents if they had at least one savings account or salary account, fixed-term account, checking account, and time and service compensation account (CTS). According to the sample obtained, 52.98% of the respondents (43,145 people) answered that they did not have an account in the financial system in 2021, while 47.02% of the respondents (38,296 people) answered that they did have an account in the financial system, showing that the majority of households in Peru do not have access to the financial system due to various information asymmetries that hinder financial inclusion (Table 2).

**Table 2:** Respondent financial inclusion, 2021.

Category	Frequency	Percentage
No	43,145	52.98%
Yes	38,296	47.02%
Total	81,441	100%

In the case of the socioeconomic characterization of households in Peru, in the area of residence, it was observed that 38.07% of the surveyed population (31,006 people) of households in Peru lived in rural areas, while the remaining 61.93% (50,435 people) lived in an urban area, showing that a slight majority of homes are located in urban areas (Table 3).

**Table 3:** Area of residence of the respondent.

Category	Frequency	Percentage
Rural	31,006	38.07%
Urban	50,435	61.93%
Total	81,441	100%

Educational levels were also examined. The analysis of the results revealed that the highest percentage of those aged 18 and above had completed their secondary studies, at 19.02%, followed by those with incomplete secondary studies at 15.54%. Meanwhile, 12.10% of the population had completed their primary level studies, and non-university higher level studies reached 5.80%, while only 5.56% of those surveyed completed university studies, as can be seen in Table 4.

**Table 4:** Educational level.

Category	Frequency	Percentage
No level	5,707	7.01%
Initial education	3,611	4.43%
Incomplete primary	17,853	21.92%
Complete primary	9,856	12.10%
Incomplete high school	12,652	15.54%
Completed secondary	15,491	19.02%
Incomplete non-university higher education	2,422	2.97%
Complete non-university higher education	4,722	5.80%
Incomplete college	3,691	4.53%
Complete university superior	4,530	5.56%
Masters Ph.D	906	1.11%
Total	81,441	100%

For the family income variable, we considered the income generated by the person per month, this being a product of their main and secondary activity in Peru. As demonstrated in Table 5, the highest percentage of respondents generated a monthly income of less than the minimum living wage of 2021, which was $251, represented by 81.91% (66,710 people). Following this, 11.56% of those surveyed (9,416 people) generated a monthly income ranging from $251 to $502, while 3.76% (3,064 people) generated an income between $503 and $753, 1.38% (1,123 people) generated an income from $754 to $1,005, and 0.61% (499 people) generated an income from $1,006 to $1,256.

**Table 5:** Monthly primary and secondary household income.

Category	Frequency	Percentage
Less than $251	66,710	81.91%
From $251 to $502	9,416	11.56%
From $503 to $753	3,064	3.76%
From $754 to $1,005	1,123	1.38%
From $1,006 to $1,256	499	0.61%
Above $1256.00	629	0.77%
Total	81,441	100%

In the case of gender, 27.82% of the people surveyed (22,655 people) were women, while 72.18% (58,786 people) were men (Table 6).

**Table 6:** Gender.

Category	Frequency	Percentage
Woman	22,655	27.82%
Man	58,786	72.18%
Total	81,441	100%

Regarding marital status, it can be seen that the highest percentage was married, being represented by 39.60% (32,254 people). On the contrary, the single population was represented by only 4.00% (3,258 people). Those in a situation of cohabitation were 33.31%, followed by widowers at 9.32%, and finally those separated or divorced at 13.77% (Table 7).

**Table 7:** Marital status.

Category	Frequency	Percentage
Single	3,258	4.00%
Widow(er)	7,593	9.32%
Separated/divorced	11,212	13.77%
Cohabitant	27,124	33.31%
Married	32,254	39.60%
Total	81,441	100%

As for socioeconomic conditions, the present investigation considered non-extreme and extreme poor people as poor and non-poor people were considered as they are in the ENAHO database. When analyzing the condition of poverty or non-poverty, it was observed that the non-poor comprised a higher percentage of 76.18%, which is equal to 62,045 people, and 23.82% were considered poor, which is equivalent to 19,396 people (Table 8).

**Table 8:** Socioeconomic status.

Category	Frequency	Percentage
Not poor	62,045	76.18%
Poor	19,396	23.82%
Total	81,441	100%

When analyzing the home ownership title, it was observed that a higher percentage of homes did not have a property title, at 49.85%, which is equal to 40,598 households; 47 79% of households had a property title, and only 2.36% were in the process of obtaining a property title (Table 9).

**Table 9:** Property titles of the households.

Category	Frequency	Percentage
Yes	38,921	47.79%
No	40,598	49.85%
In process	1922	2.36%
Total	81,441	100%

After analyzing the behavior of the determinants of financial inclusion in households, the analysis of descriptive statistics was conducted, with financial inclusion divided into two categories, namely, whether the respondents were included or not included in the financial system, with a minimum and maximum value of 1 if included in the financial system and 0 if not included. It was found that, on average, only 47.02% were included in the financial system; on average, 61.93% of those surveyed were in an urban area; on average, the respondents had incomplete secondary education; on average, the age of the respondents was between 25 and 44 years; the average family income was less than $251 per month; 72.18% were men; most were cohabitants; 23.82% were considered poor; and on average, they did not have a property title (Table 10).

In addition, an analysis of the correlation of financial inclusion and its determinants was conducted, which is detailed below:

- The relationship between area of residence and financial inclusion is direct; that is, given an increase in the probability that the person is from an urban area, then the probability of accessing credit will increase, which is corroborated by the value of Pearson ρ equal to 0.1284, corresponding to a low positive correlation.
- The relationship between educational level and financial inclusion is direct; that is, if a person has a higher educational level, then the probability of accessing credit and/or loans tends to increase, which is corroborated by the value of Pearson’s ρ equal to 0.1809, corresponding to a low positive correlation (Table 11).
- The relationship between respondent age and financial inclusion is inverse; that is, the older the person is, then the lower the probability of accessing credit and/or loans, which is corroborated by the value of Pearson’s ρ equal to −0.0049, corresponding to a low negative correlation.
- The relationship between family income and financial inclusion is direct; that is, as family income increases, then the probability of accessing credit and/or loans tends to increase, which is corroborated by the value of Pearson’s ρ equal to 0.2429, corresponding to a low positive correlation.
- The relationship between respondent gender and financial inclusion is inverse; that is, given an increase in the probability that the person is a woman, then the probability of accessing credit and/or loans tends to decrease, which is corroborated by the value of Pearson’s ρ equal to −0.0362, corresponding to a low negative correlation.
- The relationship between marital status and financial inclusion is inverse; that is, given an increase in the probability that the person is not married, then the probability of accessing credit and/or loans tends to decrease, which is corroborated by the value of Pearson’s ρ equal to −0.0235, corresponding to a low negative correlation.
- The relationship between social status and financial inclusion is indirect; that is, given an increase in the probability that the person is poor, then the probability of accessing credit and/or loans tends to decrease, which is corroborated by the value of Pearson’s ρ equal to −0.1209, corresponding to a low negative correlation.
- The existing relationship between property title and financial inclusion is indirect; that is, given an increase in the probability that the home has property title, then the probability of accessing credit and/or loans tends to decrease, which is corroborated by the value of Pearson’s ρ equal to −0.1061, corresponding to a low negative correlation.

**Table 10:** Descriptive statistics of the variables under analysis.

Variable	Mean	Standard deviation	Minimum value	Maximum value
Financial inclusion	0.4702	0.4991	0	1
Area of residence	0.6193	0.4856	0	1
Educational level	4.9770	2.4217	1	11
Age	2.8867	0.7301	1	4
Family income	1.2954	0.7708	1	6
Gender	0.7218	0.4481	0	1
Marital status	3.9519	1.1244	1	5
Social status	0.2382	0.4260	0	1
Title deed	1.5457	0.5432	1	3

**Table 11:** Correlation matrix between financial inclusion and its determinants.

Variables	Financial inclusion	Area of residence	Educational level	Age	Family income	Gender	Marital status	Social status	Title deed
Financial Inclusion	1.0000								
Area of residence	0.1284	1.0000							
Educational level	0.1809	0.3205	1.0000						
Age	−0.0049	0.072	0.0597	1.0000					
Family income	0.2429	0.1799	0.2214	−0.0219	1.0000				
Gender	−0.0362	−0.1636	−0.0314	−0.0301	−0.0098	1.0000			
Marital status	−0.0235	−0.0809	0.0026	−0.0411	0.0213	0.5411	1.0000		
Social status	−0.1209	−0.2344	−0.233	−0.1408	−0.1687	0.0732	0.0609	1.0000	
Title deed	−0.1061	−0.4564	−0.252	−0.1964	−0.1315	0.0822	0.0317	0.2132	1.0000

In this sense, to establish the determinants of financial inclusion in households in Peru, the general hypothesis test of the research was contrasted using logit-type binary regression, and the following results were obtained:

In the case of individual significance, the following null and alternate hypotheses were raised:

H:0:β:i=0 Each parameter is equal to zero (they are not statistically significant).

H:1:β:i≠0 Each parameter is different from zero (statistically significant).

Performing the individual analysis of the value of Z and its probability, the following was obtained:

- The “z” value of the area of residence is 6.26, which is greater than 2 in absolute value, and its probability is 0.000; therefore, this variable is significant at 5%, so it can explain the variability of financial inclusion.
- The “z” value of the educational level is 29.06, which is greater than 2 in absolute value, and its probability is 0.000; therefore, this variable is also significant at 5%, so it can explain the variability of financial inclusion.
- The “z” value of age is −6.08, which is greater than 2 in absolute value, and its probability is 0.000; therefore, this variable is significant at 5%, so it can explain the variability of financial inclusion.
- The “z” value of family income is 51.64, which is greater than 2 in absolute value, and its probability is 0.000; therefore, this variable is significant at 5%, so it can explain the variability of financial inclusion.
- The “z” value of gender is −2.72, which is greater than 2 in absolute value, and its probability is 0.000; therefore, this variable is significant at 5%, so it can explain the variability of financial inclusion.
- The “z” value of marital status is −4.13, which is less than 2 in absolute value, and its probability is 0.000; therefore, this variable is not significant at 5%; therefore, it cannot explain the variability of financial inclusion.
- The “z” value of the social condition is −12.36, which is greater than 2 in absolute value and its probability is 0.000; therefore, this variable is not significant at 5%; therefore, it cannot explain the variability of financial inclusion.
- The “z” value of property title is −7.98, which is greater than 2 in absolute value, and its probability is 0.000; therefore, this variable is not significant at 5%; therefore, it cannot explain the variability of financial inclusion.

Therefore, all the variables proposed in the model have individual significance, given that the value of *p* > Z is less than 0.05 or 5%.

Analyzing the global significance of area of residence, educational level, age, family income, gender, marital status, social status, and property title, the following null and alternate hypotheses were proposed:

H:0:β:i=0 All parameters have no global significance.

H:1:β:i≠0 All parameters have global significance.

Analyzing the statistical tests, the LR chi2(8) = 7,482,93, and its chi-square probability was less than 5%, thus leading us to reject the null hypothesis and accept the alternate hypothesis. Therefore, it can be indicated that they have global significance, and together, area of residence, educational level, age, family income, gender, marital status, social condition, and property title explain financial inclusion. Moreover, according to Pseudo R2, which is equal to 0.2664, 26.64% of the variation in financial inclusion is explained by area of residence, educational level, age, family income, gender, marital status, social condition, and property title (Table 11).

In this sense, for the members of households in Peru, the determinants of financial inclusion are the area of residence, educational level, age, family income, gender, marital status, social condition, and property title. Therefore, given the increase in the probability that the area of residence of the person is urban, then the probability of financial inclusion will increase by 2.76 percentage points; if the educational level of the person increases by 1, then the probability of financial inclusion will increase by 2.43 percentage points; if the respondent’s age increases by 1 year, then the probability of financial inclusion will decrease by 1.57 percentage points; if the family income increases by 1, then the probability of financial inclusion will increase by 18.57 percentage points; if the probability that the person’s gender is that of a man increases, then the probability of financial inclusion will decrease by 1.33 percentage points; if the probability that the marital status of the person changes from cohabiting to married increases, then the probability of financial inclusion will decrease by 0.80 percentage points; if the probability that the person’s socioeconomic condition is poor increases, then the probability of financial inclusion will decrease by 5.60 percentage points; and if the probability that the household member has a property title increases, then the probability of financial inclusion will decrease by 3.10 percentage points (Table 12).

## 5 Discussion

After evidencing the determinants of financial inclusion for the Peruvian case, the results obtained largely agree with the research carried out by Anaya-Narváez and Romero-Álvarez (2018). It has been found that the most influential variable for a household to have a greater probability of accessing the financial system is the educational level of the head of the household. However, in the present investigation, the income level of the head of the household was found to be an equally influential variable as the educational level, given that it includes other characteristics of the household as determinants, such as the possibility that the household receives direct state aid, which allows them to reduce the probability of being financially included. In addition, if the head of the household is a woman, then the probability of financial inclusion decreases in the same way.

**Table 12:** Logit model estimation.

Variables	Coefficient	Standard error	Z-value	*p* > Z	[95% Confidence interval]		Marginal effects
Area of residence	0.1,110	0.0177	6.2600	0.0000	0.0762	0.1457	0.0276649
Educational level	0.0974	0.0034	29.0600	0.0000	0.0909	0.1,040	0.0243091
Age	−0.0629	0.0103	−6.0800	0.0000	−0.0832	−0.0426	−0.0157034
Family income	0.7442	0.0144	51.6400	0.0000	0.7160	0.7,725	0.1857087
Gender	−0.0533	0.0196	−2.7200	0.0070	−0.0918	−0.0148	−0.0133154
Marital status	−0.0321	0.0078	−4.1300	0.0000	−0.0474	−0.0169	−0.0080221
Social status	−0.2257	0.0183	−12.3600	0.0000	−0.2615	−0.1899	−0.056066
Title deed	−0.1243	0.0156	−7.9800	0.0000	−0.1548	−0.0938	−0.0310105
Constant	−1.0114	0.0589	−17.1800	0,0000	−1.1268	−0.8960	–
Logistic regression			Number of obs		=	81,441	
Log likelihood = −52,564 694			LR chi2(8)		=	7482,93	
Marginal effects after logit			Prob > chi2		=	0.0000	
y = Pr(Financial Inclusion) (predict)			Pseudo R2		=	0.2664	
= 0.47829833							

The present investigation is also partially coincident with what was determined by Rodríguez-Raga and Riaño-Rodríguez (2016) in terms of the determinants of access to financial products, which are the level of household income, educational level, gender, and the geographic location of the household; However, these authors also considered the use of public services and the number of people per room as influential variables, which are not part of our research but may be included in future research. In this sense, we can reinforce the results obtained also with what was found by López (2023), given that the results are very similar.

On the other hand, in this scientific article, it was evidenced that gender, age, and marital status are significant variables that influence the financial inclusion process, thus contradicting what was obtained by Millán Celis and Jiménez Quitián (2016), who determined, in their research, gender and age to be non-significant. Regarding age, it was found that the older the person, the greater the probability of financial inclusion; however, this contradicts what was obtained by García-Mata and Briseño-García (2021), who indicated that older adults as having financial ignorance. In addition, they identified that having a debit card, accruing savings in the last 12 months in a financial institution, and receiving government transfers in the last 12 months allow to expand the possibility of financial inclusion in Colombia.

It is important to highlight what was found by Hoyo (2014): in terms of individual characteristics, education, income level, and gender were determinants of financial inclusion, as in our research, since they are statistically significant and have a concordant relationship in the same way. These results coincide in the same way with those of Sotomayor et al. (2020), who considered that a good economic position of the individual, a better ability to pay, having better and higher economic income, physical assets, saving money, and having Internet services contribute to access to credit from the financial system.

## 6 Conclusion

We found that 47.02% of the analyzed households in 2021 were included in the financial system. Most resided in an urban area (61.93%); most had, on average, an educational level of incomplete secondary school; the average age of the respondents was between 25 and 44 years; the average household income was less than $251 per month; 72.18% of household heads were men; most had a cohabiting marital status; 23,82were are poor; and most households did not have a property title.

The determinants of financial inclusion in households in Peru for 2021 are explained in 26.64% of the study population by area of residence, educational level, age, family income, gender, marital status, social condition, and property title. Moreover, area of residence, educational level, and family income were found to explain and positively influence financial inclusion. On the contrary, age, gender, marital status, social condition, and property title were found to negatively influence and explain financial inclusion.

Finally, if a person changes their area of residence to an urban one, increases their educational level by 1, and increases their family income level by 1, then the probability of their financial inclusion in each individual case would increase by 2.76, 2.43, and 18.57 percentage points, respectively. Moreover, if a person’s age increases by 1 year, if the probability of their gender being that of a man increases, if their marital status changes from cohabitation to marriage, if their probability of being poor increases, and if the probability of their household’s properties being titled increases, then the probability of their financial inclusion in each individual case would decrease by 1.57, 1.33, 0.80, 5.60, and 3.10 percentage points, respectively.

## Data availability statement

The raw data supporting the conclusions of this article will be made available by the authors, without undue reservation.

## Author contributions

JQ conceived and carried out the study. SA and DC contributed as studio mentors. MQ, GG, GC, LV, WQ, HM, and CR participated in the design, data analysis and writing of the scientific article. All authors reviewed and approved the research paper. All authors contributed to the article and approved the submitted version.
